# Supplementary material for: Causal Relationship between Aging and Anorexia Nervosa: A White-Matter-Microstructure-Mediated Mendelian Randomization Analysis
Source: Biomedicines. 2024 Aug 16;12(8):1874. doi: 10.3390/biomedicines12081874 (PMC11351342; doi:10.3390/biomedicines12081874)
Supplement: Supplementary file 1 [file biomedicines-12-01874-s001.zip › Supplementary Figure/Figure S4/Singe SNP plot of MR analysis for TL to ubm-b-1664.pdf]

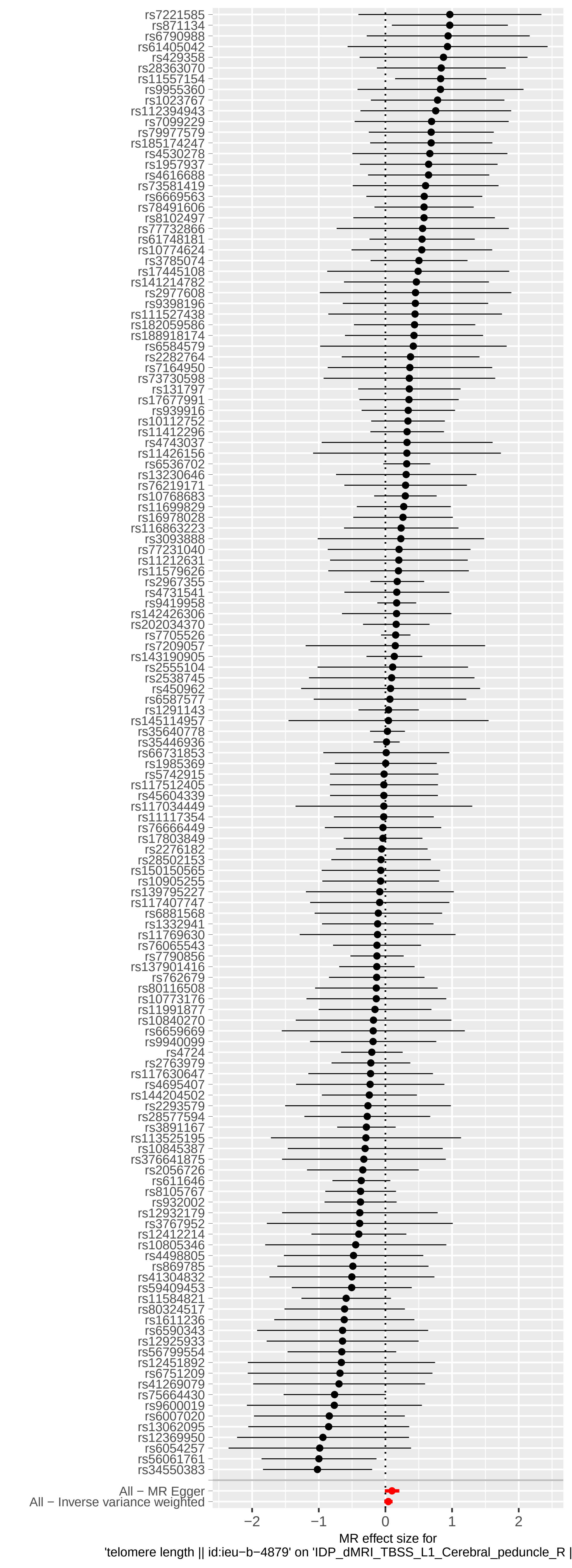
rs7221585
rs871134
rs6790988
rs61405042
rs429358
rs28363070
rs11557154
rs9955360
rs1023767
rs112394943
rs7099229
rs79977579
rs185174247
rs4530278
rs1957937
rs4616688
rs73581419
rs6669563
rs78491606
rs8102497
rs77732866
rs61748181
rs10774624
rs3785074
rs17445108
rs141214782
rs2977608
rs9398196
rs111527438
rs182059586
rs188918174
rs6584579
rs2282764
rs7164950
rs73730598
rs131797
rs17677991
rs939916
rs10112752
rs11412296
rs4743037
rs11426156
rs6536702
rs13230646
rs76219171
rs10768683
rs11699829
rs16978028
rs116863223
rs3093888
rs77231040
rs11212631
rs11579626
rs2967355
rs4731541
rs9419958
rs142426306
rs202034370
rs7705526
rs7209057
rs143190905
rs2555104
rs2538745
rs450962
rs6587577
rs1291143
rs145114957
rs35640778
rs35446936
rs66731853
rs1985369
rs5742915
rs117512405
rs45604339
rs117034449
rs11117354
rs76666449
rs17803849
rs2276182
rs28502153
rs150150565
rs10905255
rs139795227
rs117407747
rs6881568
rs1332941
rs11769630
rs76065543
rs7790856
rs137901416
rs762679
rs80116508
rs10773176
rs11991877
rs10840270
rs6659669
rs9940099
rs4724
rs2763979
rs117630647
rs4695407
rs144204502
rs2293579
rs28577594
rs3891167
rs113525195
rs10845387
rs376641875
rs2056726
rs611646
rs8105767
rs932002
rs12932179
rs3767952
rs12412214
rs10805346
rs4498805
rs869785
rs41304832
rs59409453
rs11584821
rs80324517
rs1611236
rs6590343
rs12925933
rs56799554
rs12451892
rs6751209
rs41269079
rs75664430
rs9600019
rs6007020
rs13062095
rs12369950
rs6054257
rs56061761
rs34550383
All − MR Egger
All − Inverse variance weighted
−2
−1
0
1
2
MR effect size for
'telomere length || id:ieu−b−4879' on 'IDP_dMRI_TBSS_L1_Cerebral_peduncle_R |
